# Analysis of the Refined Mean-Field Approximation for the 802.11 Protocol Model

**DOI:** 10.3390/s22228754

**Published:** 2022-11-12

**Authors:** Begoña Ispizua, Josu Doncel

**Affiliations:** 1 TECNALIA, Basque Research and Technology Alliance (BRTA), 48160 Derio, Spain; 2 Department of Mathematics, University of the Basque Country, UPV/EHU, 48940 Leioa, Spain

**Keywords:** refined mean-field approximation, 802.11 protocol model

## Abstract

Mean-field approximation is a method to investigate the behavior of stochastic models formed by a large number of interacting objects. A new approximation was recently established, i.e., the refined mean-field approximation, and its high accuracy when the number of objects is small has been shown. In this work, we consider the model of the 802.11 protocol, which is a discrete-time model and show how the refined mean-field approximation can be adapted to this model. Our results confirm the accuracy of the refined mean-field approximation when the model with N objects is in discrete time.

## 1. Introduction

### 1.1. Motivation

Stochastic population models study the behavior of a set of objects (or agents) that interact with each other. In these models, there is a finite set of states for the agents. Thus, the agents change from one state to another. An agent changes its state in a spontaneous way or by interacting with other agents. More precisely, the agents change state in a probabilistic manner that satisfies the Markov property: the probability of changing the state depends only on the present state (not on the states of the past). Under these conditions, one can construct a Markov chain; there is a wide range of tools to study its properties (see [1] for a book on this topic).

Two types of models can be distinguished according to the time at which changes in the system occur: discrete time models and continuous-time models. Discrete-time models consider that time is divided into slots and changes occur at the end of each time slot. On the other hand, continuous-time models consider that transitions take a random time, i.e., transitions occur at any time; in particular, it is very often assumed that these times are exponentially distributed. Therefore, continuous time and discrete time Markov Chains can be used to model stochastic population models in a very easy manner [1,2].

Discrete time and continuous time Markov chains that can be formulated in this setting suffer from the state-space explosion. This occurs because the state space of the Markov chain increases exponentially with the number of objects in the stochastic population model. Hence, the exact analysis of stochastic population models with a very large number of objects is an extremely difficult task to carry out (even for numerical analysis), and therefore, approximation techniques are necessary to study the behavior of these systems.

An example of an approximation technique for large stochastic population models is the mean-field approximation. The idea of the mean-field approximation is to replace a very complicated stochastic process with a simpler deterministic dynamical system, i.e., a system of (possibly non-linear) ordinary differential equations. The mean-field approximation considers that each object interacts with the average of the rest of the objects. When the number of objects is large, each object has a negligible effect on the dynamics of the whole population, and this leads to a simplification of the problem.

The refined mean-field approximation has been introduced in [3] as an approximation technique for the stochastic population model which is more accurate than the mean-field approximation. The idea of the refined mean-field approximation is to consider an additional term to the mean-field approximation which is a constant divided by the number of objects in the system. We show how this constant can be computed and illustrate the better accuracy of the refined mean-field approximation compared to the mean field approximation (even when the number of objects is small) through the following set of examples in continuous-time models: the coupon replication model, the supermarket model, the pull and push strategies model, and the bin packing model. From these examples, the authors conclude that the relative error of the refined mean field approximation is often less than 1% for systems with ten objects.

### 1.2. Contributions

In this article, we consider the model of the 802.11 protocol [4] (which falls into the class of mean field models presented in [5]) to show how the refined mean-field approximation can be adapted to discrete-time models. Let us now describe this protocol briefly. The IEEE 802.11 MAC is a set of protocols and technical standards for executing wireless local area networks, specifically the media access control set. Wi-Fi connection works use radio waves with frequency jumps, i.e., frequencies that are constantly changing, thus avoiding interference. Likewise, through Wi-Fi, the devices connected to a given network can connect to the Internet through a network access point wirelessly or via a cable. That is why the Wi-Fi consists of a compatible wireless connection between different devices. What characterizes the 802.11 protocol compared to other wireless networks is its transmission frequency of billions of waves per second.

The main contribution of this article is to investigate the accuracy of the refined mean-field approximation of the model presented in [4]. To this end, we used the rmf tool [6], which is a tool that takes the description of a stochastic model and computes numerically not only its mean-field approximations but also its refined mean-field approximation. Our results show that the accuracy of the refined mean field is much better than that of the mean-field approximation, especially when the number of objects is small.

### 1.3. Organization

The rest of the article is organized as follows. In Section 2 we put our work in the context of the existing literature, and in Section 3 we present the refined mean-field approximation. In Section 4, we describe the 802.11 protocol model and the results we have obtained. Finally, in Section 5, we provide the main conclusions of our work.

## 2. Related Work

Mean-field approximation is a widely used technique to approximate the dynamics of a stochastic system formed by a very large number of agents. For instance, it is used in the analysis of distributed queuing systems [7,8,9], in multi-agent reinforcement learning [10], neuronal networks [11], bike-sharing systems [12] and cache replacement algorithms [13]. The main advantage of the mean-field approximation is that it provides a very good approximation of stochastic models with a very large number of objects; see [14,15]. The authors in [16] showed that discrete time Markov decision processes also converge to a deterministic process when the number of objects tends to infinity.

The author in [17] showed that the mean-field approximation is 1/N-accurate when the estimation parameter is the expected value of the stochastic process (in contrast to the classical mean field approximation, which estimates the stochastic process). Using this nice accuracy property, the authors in [3] introduced a new approximation technique that refines the mean-field approximation, and they called it the refined mean-field approximation. This refinement consists of adding a constant term divided by the number of objects in the systems to the mean-field approximation. The authors in [18] developed a further approximation by considering not only the convergence to the transient state, but also a second additional term to the mean-field approximation which is proportional to the inverse of the square of the number of objects. The authors in [19] studied the refined mean-field approximation of a synchronized population.

Recently, mean field models have been also studied from the perspective of game theory [20,21,22,23,24,25,26,27,28,29,30]. These models consider that a player is under competition with the mass and assume that, given that the size of the mass is extremely big, the action taken by the player does not influence the dynamics of the mass.

## 3. The Refined Mean-Field Approximation

We consider a stochastic population process in discrete time with *N* objects, where the objects are in D<∞ possible states. Let $S_{n}^{N}\left(t\right)$ be state of the object n at time *t*. We denote by $X_{i}^{\left(N\right)}\left(t\right)$ the proportion of objects at state *i* in time *t*, i.e.,

(1) $$X_{i}^{\left(N\right)}=\frac{1}{N}\sum_{n=1}^{N}1_{S_{n}^{N}\left(t\right)=i}\;\;.$$

The vector $X^{\left(N\right)}=\left(X_{0}^{\left(N\right)},\ldots ,X_{D-1}^{\left(N\right)}\right)$ is a discrete-time Markov chain.

We assume there exists a set of vectors $L\in R^{d}$ and a set of functions $\beta_{l}$ such that the aforementioned Markov chain changes from state *x* to state x+l/N at rate $N\beta_{l}$.

We define the drift as a function such that, for every state *x*, it is associated the value f(x), where

$$f\left(x\right)=\sum_{l\in L}l\beta_{l}.$$

According to this definition, f(x)dt is the expected variation of $X^{\left(N\right)}$ at state *t* during a small interval of time dt, i.e.,

E[X(t+dt)−X(t)|X(t)=x]=f(x)dt+o(dt).

The mean-field approximation is the solution of the following ordinary differential equation:

$$\dot{x}=f\left(x\right).$$

We assume that the above equation has a single solution, and we denote it by π.

According to the result of [15], we have that

$$\lim_{N\to \infty }E\left[X^{\left(N\right)}\right]=\pi ,$$

which is true when the drift is continuous. This result means that π approximates very well the expected value of the Markov chain when the number of objects is large. Therefore, it is known in the literature as the mean-field approximation. The authors in [3] showed that there exists a constant *K* such that

$$\lim_{N\to \infty }E\left[X^{\left(N\right)}\right]=\pi +\frac{K}{N}.$$

As a consequence, the value of π+K/N (i.e., the refined mean-field approximation) provides a better approximation than the mean-field approximation π for any number of objects.

The value of *K* is expressed as a function of the Jacobian and the Hessian matrices of the drift, and the solution of a single Lyapunov equation. Therefore, as it has been shown in [3], its analytical value can be obtained for small examples. However, in more complicated models, it is required to compute this constant using numerical software. In this work, we used the rmf tool [6] to compute this constant.

In this section, we have described the refined mean-field approximation in a general setting. We refer to [3] for all the technical details and proofs of the result we have presented in this section. In the next section, however, we give an example of the 802.11 protocol model, and we analyze the refined mean-field approximation for this model.

## 4. The 802.11 Protocol Model

In this section, we concentrate of the 802.11 model. First, we present this model and how the refined mean-field approximation technique can be adapted to this discrete-time model. Then, we describe how the mean field approximation is accurate when the population size is large. Moreover, we compare the accuracy of the mean-field approximation and the refined mean-field approximation for different instances. Finally, we study how the complexity of the system influences the accuracy of the approximations under consideration in this article.

### 4.1. Model Description

**Figure 1:**
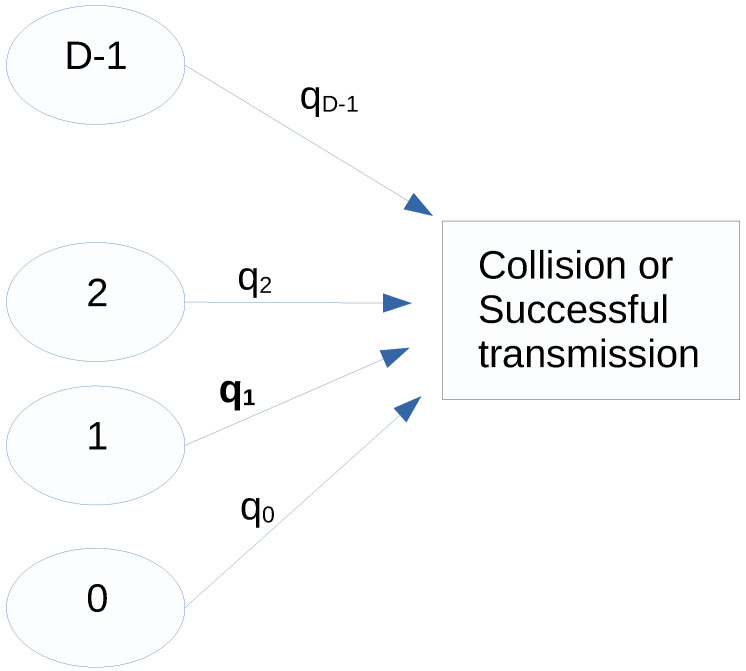
The model under study in this article.

In [4], the authors presented a discrete-time model in which N devices aim to transmit information through a common channel. It is considered that the space-set is S={0,1,…,D−1}. The state of a device represents its priority to transmit in a given time slot. A device that is at state s∈S at a given time slot tries to transmit in that time slot with probability $q_{s}/N$. This means that each state determines the probability at which a device in that state tries to transmit. Figure 1 shows the model under study in this article.

When there is a single device that tries to transmit information in one time slot, there is no collision on the transmission, and therefore, the transmission is considered to be successful. However, collision occurs when there is more that one device trying to transmit in the same time slot. In this case, none of the transmission tries are successful.

We now present the dynamics of the system, i.e., how the transmitters change from one state to another over time. When there is no collision at a given time slot, the state of the device that transmits is zero in the next time slot. On the other hand, where there is collision, the states of all the devices that try to transmit in that time slot increase by one (except for the devices at state D−1, whose state at the next time slot is zero).

We denote by $X_{i}^{\left(N\right)}\left(t\right)$ the number of devices at state *i* at time *t*, and by $X^{\left(N\right)}\left(t\right)$ the vector whose *i*-th component is $X_{i}^{\left(N\right)}\left(t\right)$. When a device is at state *i*, we define $\gamma_{i}^{\left(N\right)}\left(X\left(t\right)\right)$ as the probability that one or more devices tries to transmit during the same time slot, i.e., the probability of collision at time *t*. This can be written as follows:

(2) $$\gamma_{d}^{\left(N\right)}\left(X\left(t\right)\right)=1-\frac{\Pi_{s=0}^{D-1}{\left(1-\frac{q_{s}}{N}\right)}^{NX_{s}^{\left(N\right)}\left(t\right)}}{1-\frac{q_{d}}{N}}.$$

**Figure 2:**
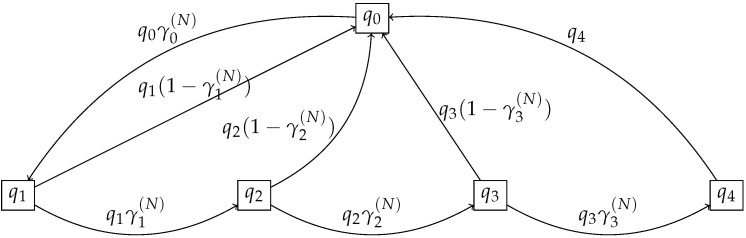
Example of the dynamics of the 802.11 model with D = 5.

In Figure 2, we illustrate the dynamics of one device when D=5.

If we assume that the duration of time steps is exponentially distributed with mean 1/N, the population process is determined by following transitions: for d=0,…,D−2

(3) $$\begin{array}{c} X\left(t\right)⟶X\left(t\right)+\frac{1}{N}\left(e_{0}-e_{d}\right)\;\mathrm{with}\;\mathrm{rate}\;Nq_{d}x_{d}\left(t\right)\left(1-\gamma_{d}^{\left(N\right)}\left(X\left(t\right)\right)\right)\\ X\left(t\right)⟶X\left(t\right)+\frac{1}{N}\left(e_{d+1}-e_{d}\right)\;\mathrm{with}\;\mathrm{rate}\;Nq_{d}x_{d}\left(t\right)\gamma_{d}^{\left(N\right)}\left(X\left(t\right)\right) \end{array}$$

and

(4) $$X\left(t\right)⟶X\left(t\right)+\frac{1}{N}\left(e_{0}-e_{D-1}\right)\;\mathrm{with}\;\mathrm{rate}\;Nq_{D-1}x_{D-1}\left(t\right),$$

where $e_{d}$ is the *d*-th element of the canonical basis.

Let us now describe each of the transitions. The transition $X\left(t\right)⟶X\left(t\right)+\frac{1}{N}\left(e_{0}-e_{d}\right)$ means that the transmission of a device at state *d* has been successful in that time slot, and therefore, the state of one device changes to zero. The transition $X\left(t\right)⟶X\left(t\right)+\frac{1}{N}\left(e_{d+1}-e_{d}\right)$ means that there has been collision since more than one device has tried to transmit. The last transition, i.e., $X\left(t\right)⟶X\left(t\right)+\frac{1}{N}\left(e_{0}-e_{D-1}\right)$, means that a device at state D−1 changes to state zero regardless of the existence of a collision or not.

When *N* tends to infinity, we have that $\gamma_{i}^{\left(N\right)}\left(x\right)$ tends to $1-e^{-\sum_{k=0}^{d-1}q_{k}x_{k}}.$ Hence, for this model, the drift is given by

$$f_{0}\left(x\right)=-q_{0}x_{0}+q_{D-1}x_{D-1}+\sum_{d=0}^{D-2}q_{d}x_{d}\left(1-\gamma \left(x\right)\right)$$

and

$$f_{d}\left(x\right)=\gamma \left(x\right)q_{d-1}x_{d-1}-q_{d}x_{d},$$

with d=1,…,D−1. This gives the following ordinary differential equation

$${\dot{x}}_{0}=f_{0}\left(x\right)$$

and

$${\dot{x}}_{d}=f_{d}\left(x\right)$$

for d=1,…,D−1. The solution of this ordinary differential equation is the mean-field approximation, and it will be our focus in the next section.

### 4.2. Mean-Field Approximation

We now concentrate on the mean-field approximation of the 802.11 model. For this case, we consider that D=5 and $q_{0}=1/2$, and $q_{d+1}=q_{d}/2$, for d=0,…,D−2. We also consider that, at time zero, all the devices are in state zero.

We studied how the stochastic process that describes the dynamics of the system approximates the mean-field approximation when the number of devices is large. We also studied the evolution of this model up to 50 s. In all the cases, we represent by a dashed line the mean-field approximation and by a solid line a trajectory of the stochastic process $X_{i}^{\left(N\right)}$, when i=0,1,2,3,4,5. Likewise, in the following figures, we plot in blue the curves that represent the mean-field approximation and the dynamics of the stochastic process when the state is 0, i.e, for $x_{0}$; in orange, we represent state 1, i.e., $x_{1}$; in green state 2, i.e., $x_{2}$; in red state 3, i.e., $x_{3}$, and finally, in violet state 4, i.e., $x_{4}$. All the figures of this section have been obtained using the command plot_ODE_vs_simulation of the rmf tool [6].

**Figure 3:**
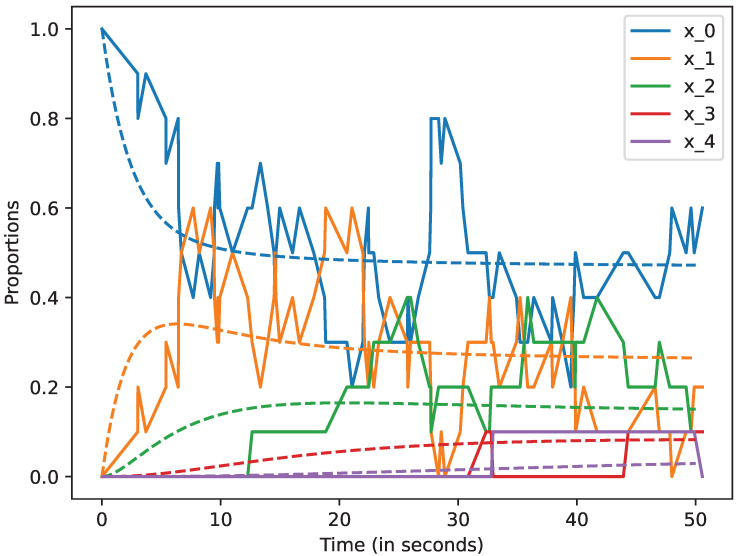
Mean-field approximation for *N* = 10 devices. Time in seconds.

In Figure 3, we consider there are N=10 devices, and can we see that the solid lines and the dashed ones are not close. This means that the mean field approximation does not provide an accurate value when N=10.

**Figure 4:**
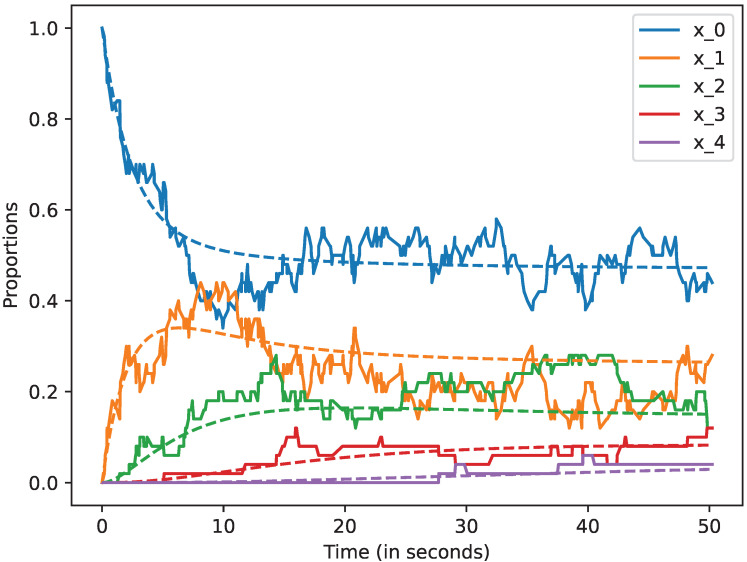
Mean-field approximation for *N* = 50 devices. Time in seconds.

Let us now consider that N=50. In Figure 4, we show that the dynamics of the stochastic processes are closer to the mean-field approximation for this case. This suggests that the accuracy of the mean-field approximation increases with *N*.

**Figure 5:**
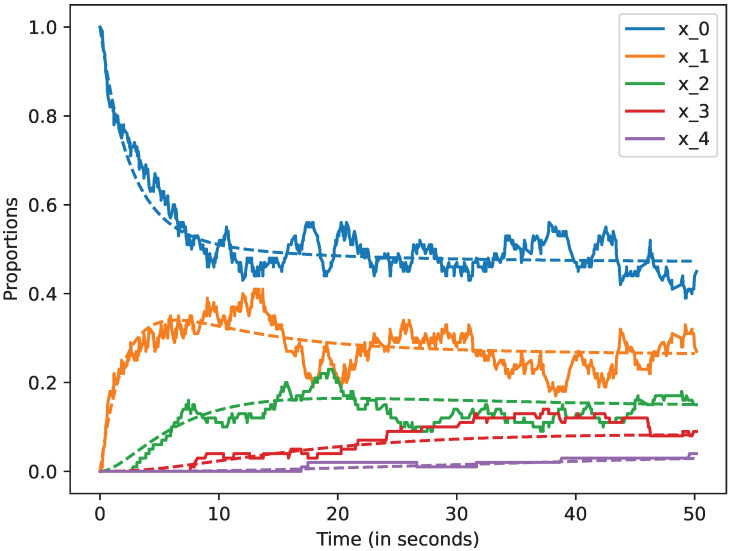
Mean-field approximation for *N* = 100 devices. Time in seconds.

There are N=100 devices in Figure 5, and we can observe that all the trajectories are quite close to the mean-field approximation. Therefore, we confirm that the accuracy of the mean-field approximation increases with *N*.

**Figure 6:**
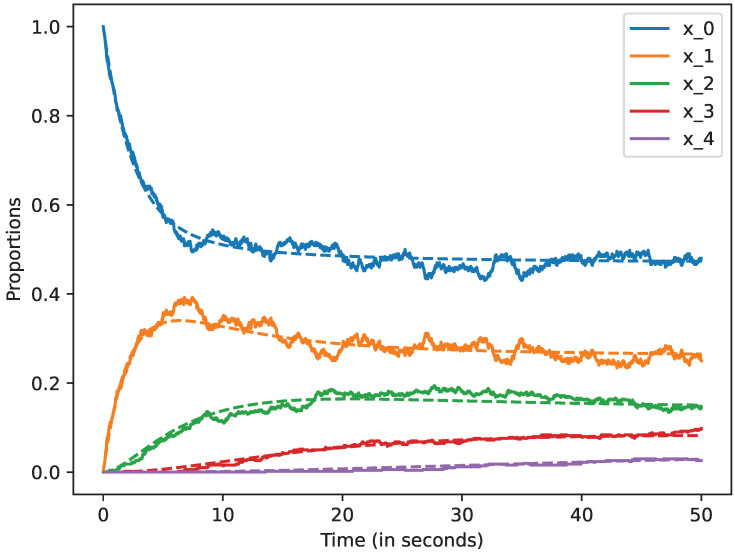
Mean-field approximation for *N* = 500 devices. Time in seconds.

**Figure 7:**
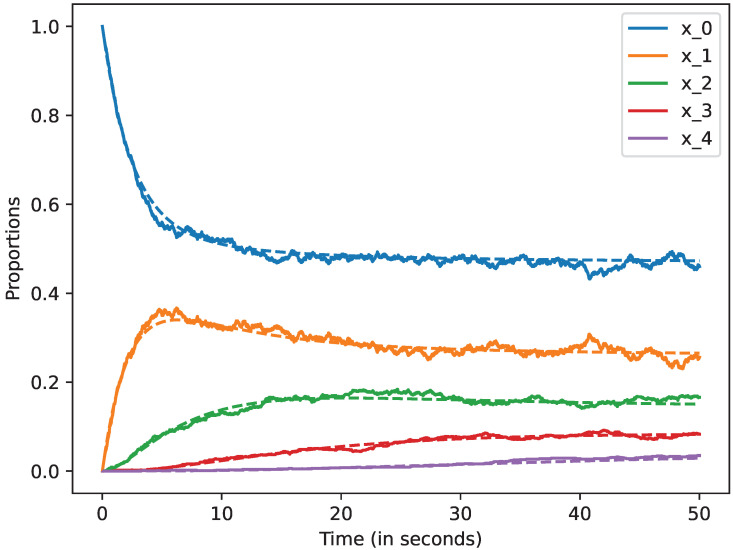
Mean-field approximation for *N* = 1000 devices. Time in seconds.

Finally, in Figure 6 and Figure 7, we consider N=500 and N=1000, respectively, and we note that the mean-field approximation is very close to the dynamics of the system in both cases. This means that the mean-field approximation is very accurate when *N* is very large.

These experiments show that the mean-field approximation is very accurate when the number of devices is very large, but when the number is devices is small, the accuracy is not good. In the following section, we will see that the refined mean-field approximation is accurate when the number of devices is small as well.

### 4.3. Refined Mean-Field Approximation

We now focus on the refined mean-field approximation. We computed numerically the mean of $X_{i}^{N}$ for different values of *N*, and we aimed to compare the obtained values with the mean-field approximation (mf) and the refined mean-field approximation (rmf) at the steady state. These values were obtained using the command compare_refinedMF of the rmf tool [6]. The results we obtained are presented in Table 1.

Our first conclusion from the simulations results we present in the above table is that the refined mean-field approximation is more accurate than the mean-field approximation. We observe that, when N=20, both approximations, as expected, are accurate. However, when N=5 and N=10, the relative error of the refined mean field approximation is much smaller. For instance, for $x_{1}$, the relative error of the refined mean-field approximation is 1% for N=5, and it is the same for N=10, whereas for the mean field approximation, the relative error for N=5 is 6%, and for N=10 it is 3%.

The tool we have used to obtain the above results gives as output the values of *K* for the refined mean-field approximation of this model. More precisely, for this model, we get the following vector:

K=[−0.0222694,0.0679408,0.0113840,−0.0236459,−0.0334095].

Taking into account that the mean-field approximation for this instance is given by

π=[0.4698643,0.2607008,0.1446480,0.0802569,0.0445300]

and the above value of the vector *K*, we conclude that, for i=0,…,4, the refined mean-field approximation for this model is given by:

- for state 0, 0.4698643−0.0222694/N
- for state 1, 0.2607008+0.0679408/N
- for state 2, 0.1446480+0.0113840/N
- for state 3, 0.0802569−0.0236459/N
- for state 4, 0.0445300−0.0334095/N

This additional term, which is a constant time 1/N, suggests an improvement on the accuracy of the refined mean-field approximation with respect to the mean-field approximation (which does not consider this additional term).

### 4.4. Accuracy vs. Complexity

We aimed to study how the complexity of the model influences the accuracy of the mean-field approximation and refined mean field approximation. For this purpose, we considered that D=15, and the rest of the parameters were the same as in the previous section. We note that, for this case, the number of transitions of the Markov chain that defines the dynamic of one object (see Figure 2 for the case D=5) increased to 30.

In the following tables (see Table 2, Table 3 and Table 4), we represent the results we obtained using the command compare_refinedMF of the rmf tool [6].

These tables show that the properties observed for D=5 still hold when D=15. This means that the refined mean-field approximation is accurate even when the complexity of the model is high.

We see that the simulation results are closer to the mean-field approximation when *N* is larger. However, we also observe that the refined mean-field approximation with *N* small provides better accuracy than the mean-field approximation.

The rmf tool also provides the value of K for each of the states. In this case, we obtained a vector K with positive and negatives values. According to the obtained values of the vector K and the mean-field approximation (see the values of the row mf in the previous tables), the refined mean-field approximation can be computed as follows:

- for state 0, 0.3861529−0.0132138/N
- for state 1, 0.2374208+0.0955549/N
- for state 2, 0.1459751+0.0449719/N
- for state 3, 0.0897510+0.0026352/N
- for state 4, 0.0551827−0.0171673/N
- for state 5, 0.0339290−0.0229363/N
- for state 6, 0.0208619−0.0219818/N
- for state 7, 0.0128282−0.0184648/N
- for state 8, 0.0078896−0.0144407/N
- for state 9, 0.0048545−0.0107968/N
- for state 10, 0.0029755−0.0078263/N
- for state 11, 0.0016016−0.0055451/N
- for state 12, 0.0005050−0.0038401/N
- for state 13, 0.0000684−0.0025717/N
- for state 14, 0.0000037−0.0016615/N

These experiments show that the refined mean-field approximation is also accurate when the complexity of the model increases.

## 5. Conclusions

We analyzed the refined mean-field approximation for the 802.11 model. First, we presented the concept of the mean-field approximation as the limit of population stochastic processes with *N* objects when the number of objects tends to infinity. The mean-field approximation consists, indeed, in calculating the solution of an ordinary differential equation, which is built using the drift of the population stochastic process with *N* objects. Then, we described the result of [17], where the author showed that the mean-field approximation is 1/N accurate when the estimation is carried out on the expected value of the stochastic process. Using this result, the authors of [3] introduced the refined mean-field approximation, which is a refinement of the mean-field approximation that is accurate not only for stochastic processes with a very large number of objects, but also when the number of objects is small.

We consider as an example of the above theory the 802.11 model. We would like to remark that using it to study the mean-field approximation has been done in [4]. Here, we went beyond the analysis presented in [4] by considering the refined mean-field approximation and comparing the accuracy of the mean-field approximation and the refined mean-field approximation when the number of objects is small. Our simulations showed that the refined mean field provides better accuracy than the mean-field approximation in all the cases, and when the number of devices is small especially, the accuracy improvement of the refinement is very large.

As future work, we aim to consider other stochastic models and compare the accuracy of the mean-field approximation and the refined mean-field approximation, both when the the number of objects is small and when the number of objects is large. We believe that the tool we have used in this article to explore the properties of the refined mean-field approximation for the 802.11 model can be very useful for this purpose.

We would also like to analyze using this tool the most recent results on the refined mean-field approximation, such as considering a second additional term (to provide an even more accurate approximation when the number of objects is small) or heterogeneity of objects in the stochastic model under consideration.

Finally, we are interesting in incorporating to the tool we have used in this article the analysis of game-theory-based stochastic models and their convergence to the derived mean field equilibrium.

## Figures and Tables

**Table 1:** Accuracy of mean-field approximation (mf) and refined mean-field approximation (rmf) when N=5,10,20.

	$x_{0}$	$x_{1}$	$x_{2}$	$x_{3}$	$x_{4}$
Sim (N = 5)	0.4453838	0.2774466	0.1346177	0.0753470	0.0344656
Sim (N = 10)	0.4680003	0.2704071	0.1522602	0.0667931	0.0255959
Sim (N = 20)	0.4703394	0.2569733	0.1484465	0.0731954	0.0410477
mf	0.4698643	0.2607008	0.1446480	0.0802569	0.0445300
Rmf (N = 5)	0.4653996	0.2744316	0.1469139	0.0754686	0.0377863
Rmf (N = 10)	0.4672855	0.2676632	0.1458856	0.0779468	0.0412189
Rmf (N = 20)	0.4687482	0.2641335	0.1452145	0.0790598	0.0428440

**Table 2:** Accuracy of mean-field approximation (mf) and refined mean-field approximation (rmf) when N=5,10,20 for $x_{0},\ldots ,x_{4}$.

	$x_{0}$	$x_{1}$	$x_{2}$	$x_{3}$	$x_{4}$
Sim (N = 5)	0.3544870	0.2331678	0.1355447	0.0603250	0.0447286
Sim (N = 10)	0.3797964	0.2525756	0.1514744	0.0833863	0.0451816
Sim (N = 20)	0.3876362	0.2416717	0.1481701	0.0845466	0.0490803
mf	0.3861529	0.2374208	0.1459751	0.0897510	0.0551827
Rmf (N = 5)	0.3835102	0.2565318	0.1549694	0.0902781	0.0417492
Rmf (N = 10)	0.3797964	0.2525756	0.1514744	0.0833863	0.0451816
Rmf (N = 20)	0.3812561	0.2462875	0.15092110	0.0908213	0.0512771

**Table 3:** Accuracy of mean-field approximation (mf) and refined mean-field approximation (rmf) when N=5,10,20 for $x_{5},\ldots ,x_{9}$.

	$x_{5}$	$x_{6}$	$x_{7}$	$x_{8}$	$x_{9}$
Sim (N = 5)	0.0286355	0.0260564	0.0168371	0.0493625	0.0192964
Sim (N = 10)	0.0382880	0.0100355	0.0058542	0.0005560	0.0017155
Sim (N = 20)	0.0306790	0.0192795	0.0067368	0.0008361	0.0238537
mf	0.0339290	0.0208619	0.0128282	0.0078896	0.0048545
Rmf (N = 5)	0.0293418	0.0164655	0.0091352	0.0050014	0.0026952
Rmf (N = 10)	0.0323172	0.0191875	0.0113629	0.0067129	0.0039576
Rmf (N = 20)	0.0327822	0.0197628	0.0119050	0.0071675	0.0043147

**Table 4:** Accuracy of mean-field approximation (mf) and refined mean-field approximation (rmf) when N=5,10,20 for $x_{10},\ldots ,x_{14}$.

	$x_{10}$	$x_{11}$	$x_{12}$	$x_{13}$	$x_{14}$
Sim (N = 5)	0.0010142	0.0006991	0.0000025	0.0000007	0.0000001
Sim (N = 10)	0.0019843	0.0010403	0.0000352	0.0000012	0.0000001
Sim (N = 20)	0.0022454	0.0012491	0.0001233	0.0000126	0.0000009
mf	0.0029755	0.0016016	0.0005050	0.0000684	0.0000037
Rmf (N = 5)	0.0014102	0.0004926	0.0000630	0.0000046	0.0000003
Rmf (N = 10)	0.0023148	0.0011025	0.0000134	0.0000099	0.0000007
Rmf (N = 20)	0.0025841	0.0013244	0.0003130	0.0000160	0.0000011
